# Transcranial Doppler Combined With Quantitative Electroencephalography Brain Function Monitoring for Estimating the Prognosis of Patients With Posterior Circulation Cerebral Infarction

**DOI:** 10.3389/fneur.2021.600985

**Published:** 2021-05-17

**Authors:** Yanting Cao, Xiaonan Song, Lijuan Wang, Yajie Qi, Ying Chen, Yingqi Xing

**Affiliations:** ^1^Department of Vascular Ultrasonography, Xuanwu Hospital, Capital Medical University, Beijing, China; ^2^Department of Neurology, The First Hospital of Jilin University, Changchun, China; ^3^Department of Neurology, Linyi People’s Hospital, Linyi, China; ^4^Beijing Diagnostic Center of Vascular Ultrasound, Beijing, China; ^5^Center of Vascular Ultrasonography, Beijing Institute of Brain Disorders, Collaborative Innovation Center for Brain Disorders, Capital Medical University, Beijing, China

**Keywords:** transcranial Doppler, quantitative electroencephalography, cerebral infarction, brain function monitoring, prognosis

## Abstract

Posterior circulation cerebral infarction (PCCI) can lead to deceased infratentorial cerebral blood flow (CBF) and metabolism. Neural activity is closely related to regional cerebral blood flow both spatially and temporally. Transcranial Doppler (TCD) combined with quantitative electroencephalography (QEEG) is a technique that evaluates neurovascular coupling and involves synergy between the metabolic and vascular systems. This study aimed to monitor brain function using TCD-QEEG and estimate the efficacy of TCD-QEEG for predicting the prognosis of patients with PCCI. We used a TCD-QEEG recording system to perform quantitative brain function monitoring; we recorded the related clinical variables simultaneously. The data were analyzed using a Cox proportional hazards regression model. Receiver-operating characteristic (ROC) curve analysis was used to evaluate the cut-off for the diastolic flow velocity (VD) and (delta + theta)/(alpha + beta) ratio (DTABR). The area under the ROC curve (AUROC) was calculated to assess the predictive validity of the study variables. Forty patients (aged 63.7 ± 9.9 years; 30 men) were assessed. Mortality at 90 days was 40%. The TCD indicators of VD [hazard ratio (HR) 0.168, confidence interval (CI) 0.047–0.597, *p* = 0.006] and QEEG indicators of DTABR (HR 12.527, CI 1.637–95.846, *p* = 0.015) were the independent predictors of the clinical outcomes. The AUROC after combination of VD and DTABR was 0.896 and showed better predictive accuracy than the Glasgow Coma Scale score (0.75), VD (0.76), and DTABR (0.781; all *p* < 0.05). TCD-QEEG provides a good understanding of the coupling mechanisms in the brain and can improve our ability to predict the prognosis of patients with PCCI.

## Introduction

Stroke is a leading cause of mortality and disability (1). Cerebral infarction accounts for 87% of the cases of stroke (2), and the mortality and disability rates are higher for patients with posterior circulation cerebral infarction (PCCI), especially those who require neurological intensive care, than for those with anterior circulation stroke (3, 4). Cerebral ischemia leads to a decrease in cerebral blood flow (CBF) and metabolism. Early metabolic changes in the brains of patients with cerebral infarction provide important information concerning diagnosis and treatment (5). After the onset of ischemic stroke, CBF is disrupted throughout the affected region of the brain; this sharp reduction in blood flow results in deficient adenosine triphosphate levels and subsequent ionic disruption and metabolic failure, which progresses within minutes to neuronal necrosis (6). The close temporospatial relationship between neural activity and regional CBF is known as neurovascular coupling (7, 8).

The flow dynamics of intracranial arterial blood are assessed by transcranial Doppler (TCD) and the physiological parameters of blood flow by the ultrasonic Doppler effect (9, 10). Distal flow in the posterior circulation is a robust indicator of the risk of vertebrobasilar artery stroke in patients with symptomatic atherosclerotic vertebrobasilar artery occlusive disease (11). This suggests that hypoperfusion is a key underlying mechanism and that distal hemodynamic compromise provides valuable prognostic information. TCD provides supporting evidence for this hemodynamic mechanism. Electroencephalography (EEG) can sensitively reflect the deterioration of metabolism and disturbance of neural activity after a decrease in CBF (12, 13). Quantitative EEG (QEEG) is a digital analysis method that transforms the electroencephalogram into power spectra by Fast Fourier transform; this compresses the EEG data, thereby making review more efficient. When normal CBF decreases to ~25–35 mL/100 g/min, the electroencephalogram first loses faster frequencies, and when CBF decreases to ~17–18 mL/100 g/min, slower frequencies gradually increase (14). Sheorajpanday et al. used QEEG to evaluate patients with PCCI and found that PCCI could be detected early with QEEG and that the pairwise-derived brain symmetry index (pdBSI) was an independent predictor of definite stroke in patients with PCCI (15). TCD-QEEG is a novel non-invasive neurovascular coupling technique that can evaluate the synergy between the metabolic and vascular systems and can be performed at the bedside.

A study by Chen et al. reported that the prognostic accuracy of TCD-QEEG was statistically superior to that of any single clinical or neurophysiological assessment in patients with intracerebral hemorrhage (16). However, studies have not reported on the prognostic value of TCD combined with QEEG for patients with PCCI. Given the high mortality and morbidity among patients with PCCI in neurological intensive care units, it seems important to develop a method that could be used to predict patient outcomes. This study aimed to combine TCD and QEEG for monitoring of brain function and estimation of the prognosis of patients with PCCI.

## Materials and Methods

### Patients

The study protocol was approved by the Ethics Committee of the First Hospital of Jilin University, China and was conducted in accordance with the tenets of the Declaration of Helsinki. Consecutive patients with PCCI who were admitted to the Neurological Intensive Care Unit (NICU) between July 2018 and July 2019 were prospectively enrolled. Written informed consent was obtained from the immediate family members before inclusion in the study. The inclusion criteria were admission within ≤72 h of onset, PCCI diagnosed on clinical examination, magnetic resonance imaging (MRI), and age ≥18 years. The exclusion criteria were as follows: presence of cerebral hemorrhage; stroke induced by a brain tumor, moyamoya disease, or hematological disease; previous ischemic or hemorrhagic cerebrovascular disease; marked environmental disturbance, such as severe hypoxia (<50 mg/dL) or hyperglycemia (>400 mg/dL); recanalization therapy; and use of a central nervous system depressant, such as a sedative, antipsychotic, or antiepileptic drug. Twenty healthy controls matched for age and sex (58.1 ± 5.1 years, 15 men) were also enrolled.

### Clinical Data

We recorded and analyzed the following variables: demographic characteristics (patient age and sex); risk factors (hypertension, diabetes mellitus, atrial fibrillation, myocardial infarction, smoking, and alcohol consumption) using the definitions of hypertension and diabetes mellitus reported by Song et al. (17); Glasgow Coma Scale (GCS) and Full Outline of UnResponsiveness (FOUR) scores on admission; vital signs (systolic blood pressure, diastolic blood pressure, and heart rate); laboratory findings (serum levels of potassium, calcium, sodium, and glucose, white blood cell count, platelet count, and prealbumin levels); and ejection fraction. In previous studies, GCS score, FOUR scores, atrial fibrillation, and other factors have been determined as significant factors for the prognosis of patients with PCCI (18–20). Conventional TCD (EMS-9PB; Delica Medical, Shenzhen, China) and carotid ultrasound (CX50; Philips, Andover, MA, USA) were performed to diagnose stenosis or occlusion of the vessels involved in the posterior circulation in all patients. The clinical outcome was assessed using the five-point Glasgow Outcome Scale score at 90 days after ictus.

### TCD-QEEG Measurements

TCD combined with QEEG monitoring (Nicolet EEG Monitor; Natus Medical, Pleasanton, CA, USA) was performed with the patient in the supine position. TCD was performed using 2-MHz pulsed-wave Doppler probes fixed to each temporal window with a helmet. We measured bilateral posterior cerebral arteries and optimal posterior cerebral artery signals were acquired at a depth of 60–75 mm bilaterally. When the temporal acoustic bone window in TCD was inadequate, we took the probe off the helmet and manually fixed it to a pillow window and measured the velocity of the ipsilateral vertebral blood flow (at a depth of 50–80 mm) because both the posterior cerebral artery and vertebral artery reflect the hemodynamics of the posterior circulation. QEEG was recorded according to the 16-electrode system installed in the international 10-20 system using bipolar longitudinal Fp1-F3, Fp2-F4, F3-C3, F4-C4, C3-P3, C4-P4, P3-O1, P4-O2, F7-T3, F8-T4, T3-T5, and T4-T6 with 0.5-Hz low frequency and 35-Hz high frequency filters. The band resolution was 0.25 Hz and the sampling frequency was 500 Hz. The impedance of each lead and electrode was maintained at <5 kΩ. Patients in the healthy control group remained awake with eyes closed during the procedure. The specific monitoring time took place at 9:00 A.M. or 2:00 P.M. on the second day after admission. Each patient was monitored only once. The data were recorded for over 30 min until a stable recording was established; the recorded data were stored for further analysis.

### Data Analysis

The data for each patient were analyzed in a blinded manner. The systolic flow velocity (VS), diastolic flow velocity (VD), mean velocity (VM), and pulsatility index (PI) from the left and right hemispheres were recorded with TCD. VM was calculated as (VS–VD)/3 + VD and PI as (VS–VD)/VM. Considering that the lesions in the vessels of the posterior circulation are mostly bilateral, we averaged the TCD data between the two hemispheres. All segments of artifact-free EEG were selected and quantitatively analyzed offline to compute the relative power using Fast Fourier transform for each electrode over the 1–35 Hz range, as follows: relative delta power (0.5–4 Hz), relative theta power (4–8 Hz), relative alpha power (8–13 Hz), and relative beta power (13–35 Hz). We recorded the following QEEG parameters: delta/alpha ratio (DAR), (delta + theta)/(alpha + beta) ratio (DTABR), alpha/beta ratio, brain symmetry index (BSI), alpha variability, spectral entropy, 95% spectral edge frequency, gross energy, median frequency, peak frequency, ambulatory EEG, envelope analysis, and delta ratio.

### Statistical Analysis

The data for the univariate analysis are reported as the mean ± standard deviation for normally distributed variables and as the median (interquartile range) for non-normally distributed variables. Categorical variables are presented as percentages. The Student's *t*-test and median two-sample test were used to examine normally distributed variables, and non-parametric Wilcoxon (Kruskal-Wallis) analysis of variance was used for non-normally distributed variables. Categorical variables were compared using the chi-squared test. Risk factors with a *p*-value < 0.05 in univariate analysis were included in a multivariate Cox proportional hazards regression forward model to analyze the hazard ratio (HR) for mortality. The survival rates were assessed by Kaplan-Meier analysis using log-rank tests to evaluate the significance of the multivariate models and compare the survival curves. Receiver-operating characteristic (ROC) curve analysis was used to evaluate the cut-off values for VD and DTABR, and the area under the ROC curve (AUROC) was used to assess the predictive ability of the variables. The ROC curves were compared using DeLong's test. All statistical tests were two-tailed, and a *p*-value < 0.05 was considered statistically significant. All statistical analyses were performed using SPSS version 17.0 (SPSS Inc, Chicago, IL, USA), GraphPad Prism 8.0 (GraphPad Software, La Jolla, CA, USA), and MedCalc 19.0.7 (MedCalc Software, Mariakerke, Belgium).

## Results

### Basic Data

Forty-six patients with PCCI were enrolled. Three patients were excluded because of signal artifacts and three patients because of lack of follow-up. Finally, we enrolled 40 patients, 16 (40%) of whom died during the 90-day follow-up period. The median age was 63.7 ± 9.9 years and 30 (75%) of the patients were male.

Combined TCD and carotid ultrasound yielded the following results: normal (history of atrial fibrillation or myocardial infarction, *n* = 5), unilateral vertebral artery stenosis or occlusion (*n* = 12), and bilateral vertebral and/or basilar artery occlusion (*n* = 23). As TOAS types of occlusion, 35 patients (87.5%) with PCCI showed an atherosclerotic occlusion of the great arteries and five patients (12.5%) with PCCI showed a cardiogenic occlusion. The only variables associated with mortality were the GCS (*p* = 0.001) and FOUR scores (*p* = 0.008). There was no statistically significant between-group difference in patient demographics, risk factors, vital signs, laboratory findings, or ejection fraction (*p* > 0.05; Table 1).

**Table 1:** Clinical data at baseline.

**Characteristics**	**All patients (*n* = 40)**	**Non-survivors (*n* = 16)**	**Survivors (*n* = 24)**	***P*-value**
**Demographic variables**				
Age (years), mean (SD)	63.7 (9.9)	61.2 (8.2)	65.3 (10.7)	0.203
Male, *n* (%)	30 (75)	14 (87.5)	16 (66.7)	0.264
**Risk factors**, ***n*** **(%)**				
Hypertension	34 (85)	12 (75)	22 (91.7)	0.32
Diabetes mellitus	11 (27.5)	5 (31.3)	6 (25)	0.942
Atrial fibrillation	8 (20)	2 (12.5)	6 (25)	0.572
Myocardial infarction	4 (10)	1 (6.3)	3 (12.5)	0.914
Smoking	21 (52.5)	9 (56.3)	12 (50)	0.698
Drinking	19 (47.5)	10 (62.5)	9 (37.5)	0.121
GCS score, *n* (%)				0.001
GCS score < 8	13 (32.5)	10 (76.9)	3 (23.1)	
GCS score > 8	27 (67.5)	6 (22.2)	21 (77.8)	
FOUR score, median (IQR)	15 (11–16)	11.5 (9–15.75)	16 (14.25–16)	0.008
**Vital signs**				
SBP (mmHg), mean (SD)	157.9 (19.8)	159.9 (19.1)	156.6 (20.6)	0.618
DBP (mmHg), mean (SD)	84.4 (12.1)	84.8 (11.9)	84.2 (12.5)	0.871
HR (bpm), median (IQR)	81.5 (74.3–96.3)	87 (75.8–97)	80 (74–94)	0.334
**Laboratory findings**				
WBC (× 10^9^/L), median (IQR)	9.8 (8.6–13.4)	9.5 (8.9–12.9)	10.2 (8.6–15.6)	1
Platelets (× 10^9^/L), mean (SD)	206 (67)	211 (67)	203 (68)	0.723
Glucose (mmol/L), median (IQR)	6.7 (5.3–9.5)	7 (5.9–9.7)	5.9 (5.1–9.5)	0.23
Potassium (mmol/L), mean (SD)	3.82 (0.56)	3.82 (0.61)	3.83 (0.53)	0.946
Calcium (mmol/L), mean (SD)	2.19 (0.16)	2.22 (0.2)	2.18 (0.13)	0.45
Sodium (mmol/L), mean (SD)	140.1 (4.6)	139.5 (4.7)	140.5 (4.5)	0.496
PA (g/L), median (IQR)	0.22 (0.18–0.25)	0.23 (0.17–0.24)	0.22 (0.18–0.25)	0.761
EF (%), median (IQR)	58 (57–60)	59 (57–60)	58 (57–59)	0.386

*DBP, diastolic blood pressure; EF, ejection fraction; FOUR, Full Outline of UnResponsiveness; GCS, Glasgow Coma Scale; HR, heart rate; IQR, interquartile range; PA, prealbumin; SBP, systolic blood pressure; SD, standard deviation; WBC, white blood cell count*.

### Evaluation of Brain Function With TCD-QEEG

**Figure 1:**
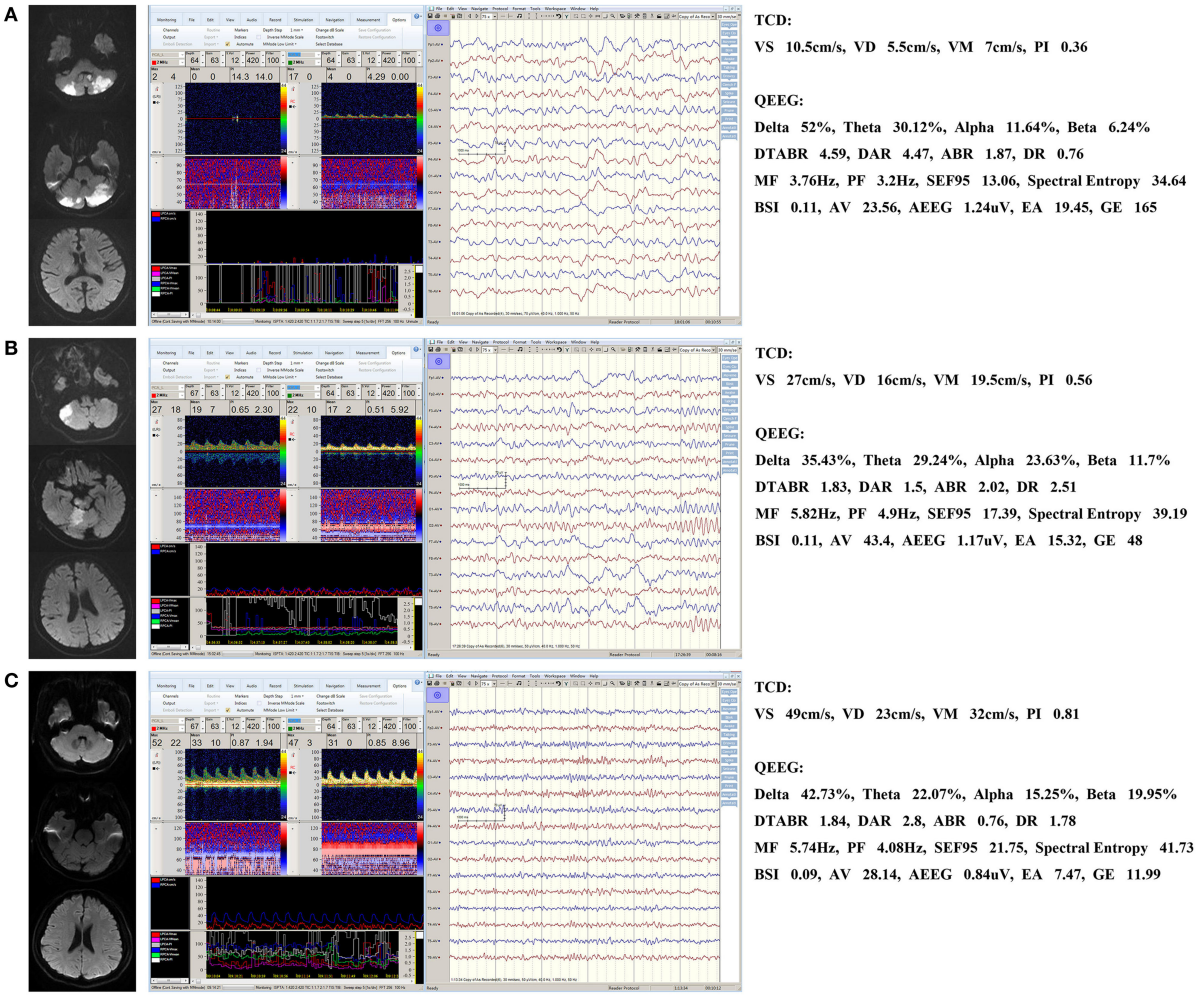
Representative patients. **(A)** QEEG for a non-survivor showing a significant increase in the slower delta frequency band and a significant decrease in the faster alpha frequency band. MF and spectral entropy decreased and DTABR and DAR (but not BSI) increased. TCD shows that the VD, VS, and VM decreased. **(B)** QEEG and TCD for a survivor showing similar changes that are not as significant as those seen in a. BSI did not increase either. **(C)** Normal QEEG and TCD in a healthy control patient. DTABR, (delta + theta)/(alpha + beta) ratio; DAR, delta/alpha ratio; BSI, brain symmetry index; MF, median frequency; VS, systolic flow velocity; VM, mean flow velocity; VD, diastolic flow velocity; PI, pulsatility index; TCD, transcranial Doppler; QEEG, quantitative electroencephalography.

**Figure 2:**
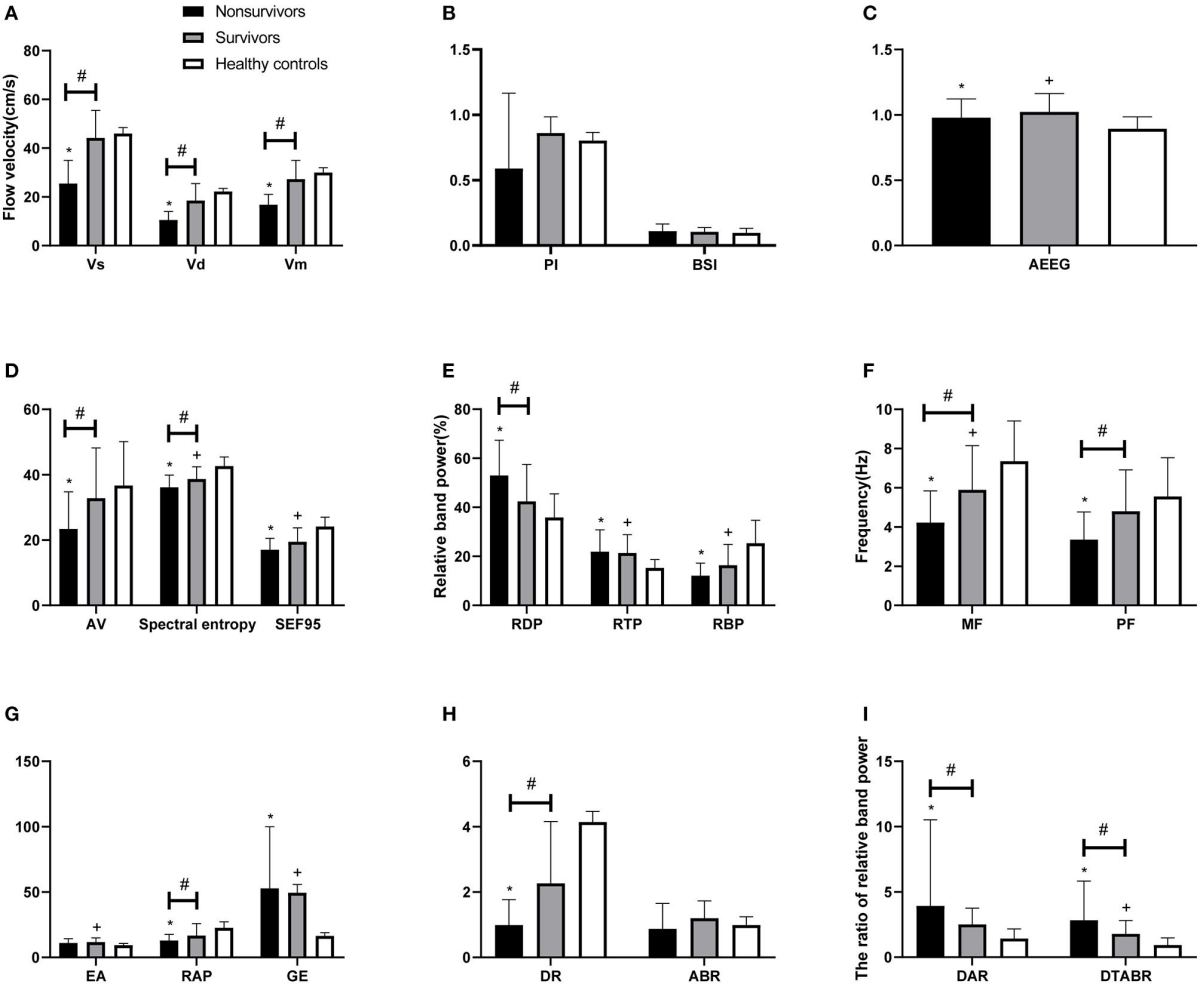
Comparison of TCD and QEEG parameters between patients with PCCI and healthy controls. **(A)** Systolic flow velocity (VS), diastolic flow velocity (VD), mean flow velocity (VM); **(B)** pulsatility index (PI), brain symmetry index (BSI); **(C)** amplitude-integrated EEG (AEEG); **(D)** alpha variability (AV), spectral entropy, 95% spectral edge frequency (SEF95); **(E)** relative band power of delta, theta, beta; **(F)** median frequency (MF), peak frequency (PF); **(G)** envelope analysis (EA), relative band power of alpha, gross energy (GE); **(H)** delta ratio (DR), alpha/beta ratio (ABR); **(I)** delta/alpha ratio (DAR), (delta + theta)/(alpha + beta) ratio (DTABR). ^#^*P* < 0.05 for non-survivors vs. survivors; *P < 0.05 for non-survivors vs. healthy controls; ^+^*P* < 0.05 for survivors vs. healthy controls.

Figure 1 shows the MRI and TCD-QEEG findings in representative patients. Among the relevant TCD indicators, a lower VS (*p* = 0.014), VD (*p* = 0.001), and VM (*p* = 0.012) were associated with mortality. There was no significant difference in VS, VD, or VM between survivors and healthy controls or in PI among any of the groups (all *p* > 0.05; Table 2, Figures 2A,B).

**Table 2:** Transcranial Doppler and quantitative electroencephalographic parameters.

	**Non-survivors (*n* = 16)**	**Survivors (*n* = 24)**	**Healthy controls (*n* = 20)**
**TCD parameters**			
VS (cm/s) median (IQR)	25.5 (13.13–34.25)^#^^*^	44.25 (22.38–56.25)	46 (42.88–51.13)
**VD**, ***n*** **(%)**			
VD ≤ 14.5 (cm/s)	13 (81)^#^^*^	7 (30)	1 (5)
VD > 14.5 (cm/s)	3 (19)^#^^*^	17 (70)	19 (95)
VM (cm/s) median (IQR)	16.75 (8–20.5)^#^^*^	27.25 (14.38–35.75)	30 (27.5–32.38)
PI, median (IQR)	0.59 (0.38–1.12)	0.86 (0.65–1)	0.8 (0.77–0.87)
**QEEG parameters**			
BSI, median (IQR)	0.111 (0.09–0.16)	0.106 (0.093–0.146)	0.097 (0.09–0.132)
AEEG (μV), mean (SD)	0.98 (0.14)^*^	1.02 (0.14)^+^	0.89 (0.09)
AV, mean (SD)	23.4 (11.3)^#^^*^	32.8 (15.4)	36.7 (13.4)
Spectral entropy, mean (SD)	36.1 (3.8)^#^^*^	38.7 (3.7)^+^	42.6 (2.8)
SEF95 (Hz), mean (SD)	17 (3.6)^*^	19.5 (4.3)^+^	24.2 (2.9)
RDP (%), mean (SD)	53 (14.4)^#^^*^	42.4 (15.1)	35.8 (9.6)
RTP (%), mean (SD)	21.9 (8.9)^*^	21.4 (7.5)^+^	15.3 (3.4)
RBP (%), mean (SD)	12.1 (5.2)^*^	16.4 (8.5)^+^	25.3 (9.4)
MF (Hz), mean (SD)	4.22 (1.62)^#^^*^	5.89 (2.26)^+^	7.35 (2.05)
PF (Hz), mean (SD)	3.35 (1.41)^#^^*^	4.79 (2.12)	5.55 (1.98)
EA, median (IQR)	11.1 (8–14.1)	11.6 (9.8–15.2)^+^	9.3 (7.6–11.1)
RAP (%), median (IQR)	12.9 (6.6–17)^#^^*^	16.7 (13.2–26.9)	22.6 (17.2–28.4)
GE, median (IQR)	52.8 (35.3–97.3)^*^	49.4 (33–61.6)^+^	16.4 (11.6–20.1)
DR, median (IQR)	0.99 (0.43–1.69)^#^^*^	2.26 (0.96–4.71)	4.14 (1.8–5.14)
ABR, median (IQR)	0.87 (0.66–1.58)	1.2 (0.81–1.79)	0.99 (0.64–1.34)
DAR, median (IQR)	3.94 (2.89–9.29)^#^^*^	2.52 (1.09–3.77)	1.43 (1.17–2.58)
**DTABR**, ***n*** **(%)**			
DTABR ≤ 2	1 (6)^#^^*^	15 (62.5)^+^	18 (90)
DTABR > 2	15 (94)^#^^*^	9 (37.5)^+^	2 (10)

*ABR, alpha/beta ratio; AEEG, ambulatory electroencephalography; AV, alpha variability; BSI, brain symmetry index; DAR, delta/alpha ratio; DR, delta ratio; DTABR, (delta + theta)/(alpha + beta) ratio; EA, envelope analysis; GE, gross energy; IQR, interquartile range; MF, median frequency; PF, peak frequency; PI, pulsatility index; RAP, relative alpha power; RBP, relative beta power; RDP, relative delta power; RTP, relative theta power; SD, standard deviation; SEF95, 95% spectral edge frequency; VD, diastolic flow velocity; VM, mean flow velocity; VS, systolic flow velocity*.

# *p < 0.05 for non-survivors vs. survivors*,

* *p < 0.05 for non-survivors vs. healthy controls*,

+ *p < 0.05 for survivors vs. healthy controls*.

Among the QEEG-relevant indicators, a higher relative delta power (*p* = 0.033), higher DAR (*p* = 0.027), higher DTABR (*p* < 0.0001), lower alpha variability (*p* = 0.043), lower relative alpha power (*p* = 0.027), lower delta ratio (*p* = 0.02), lower spectral entropy (*p* = 0.042), lower median frequency (*p* = 0.015), and lower peak frequency (*p* = 0.022) were associated with mortality. There were significant differences in QEEG, relative theta and beta power, spectral entropy, 95% spectral edge frequency, DTABR, gross energy, and median frequency (all *p* < 0.05) between the patients with PCCI and healthy controls. There was no significant difference in the BSI or alpha/beta ratio (all *p* > 0.05) among any of the groups (Table 2, Figures 2B–I).

### Multivariate Analysis

All variables with *p* ≤ 0.05 in the univariate analysis were used in the Cox proportional hazards model with mortality at 90 days as the dependent variable. Only DTABR (HR 12.527, 95% CI 1.637–95.846, *p* = 0.015) and VD (HR 0.168, 95% CI 0.047–0.597, *p* = 0.006) were independent predictors of mortality at 90 days.

### Comparison of the Survival Curves and ROC Curves

**Figure 3:**
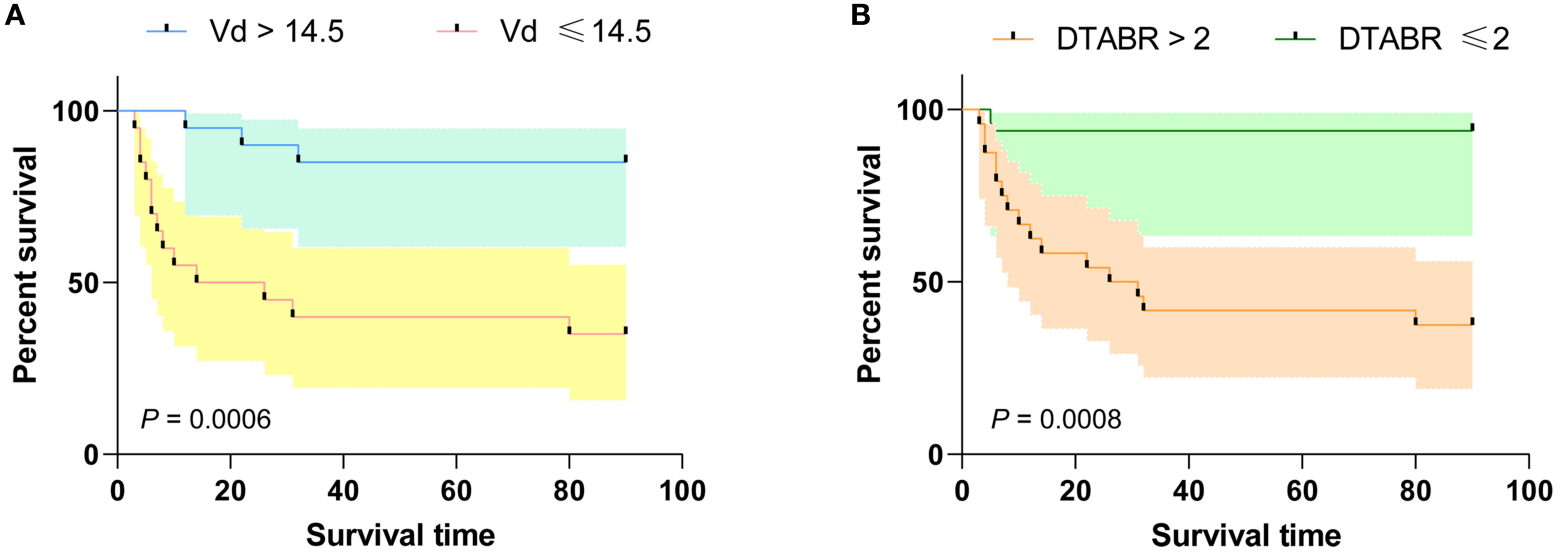
Kaplan-Meier survival curves for the training cohort. **(A)** Kaplan-Meier survival curves for VD; **(B)** Kaplan-Meier survival curves for DTABR. Patients with a VD ≤ 14.5 had a higher risk of death than those with a VD > 14.5; the 90-day survival rate was lower in patients with a DTABR > 2 than in patients with a DTABR ≤ 2. VD, diastolic flow velocity; DTABR, (delta + theta)/(alpha + beta) ratio.

ROC curve analysis, performed to evaluate the cut-off points for VD and DTABR, revealed that the optimal VD cut-off for prediction of mortality risk was 14.5 cm/s. Patients with a VD ≤ 14.5 cm/s had a significantly greater risk of death than those with a VD > 14.5 cm/s (65 vs. 15%, *p* = 0.001). Median survival in patients with a VD ≤ 14.5 cm/s was 20 days. The optimal DTABR cut-off for predicting the mortality risk was 2. The 90-day survival rate was 60% (95% CI 49.05–72.45) for all patients but was lower in patients with a DTABR > 2 than in those with a DTABR ≤ 2 (37.5 vs. 93.8%, *p* = 0.001). Median survival in those with a DTABR > 2 was 28.5 days (Figure 3).

**Figure 4:**
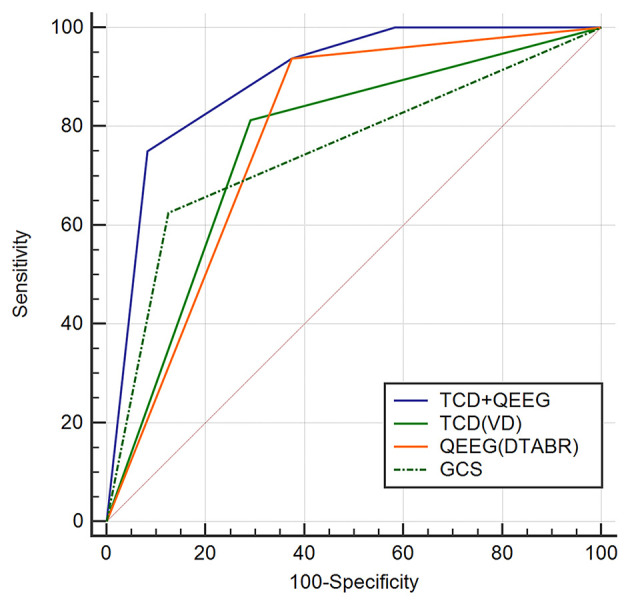
Comparison of ROC curves (AUROC) to predict the outcome between the four models in this cohort. Glasgow Coma Scale (GCS), AUROC 0.75 (0.588–0.873); diastolic flow velocity (VD), AUROC 0.76 (0.599–0.881); (delta + theta)/(alpha + beta) ratio (DTABR), AUROC 0.781 (0.622–0.896); transcranial Doppler (TCD) + quantitative EEG (QEEG), AUROC 0.896 (0.758–0.97). *P* < 0.05 for TCD (VD) + QEEG (DTABR) comparison with GCS, VD (independent predictor of TCD), and DTABR (independent predictor of QEEG).

To determine whether TCD combined with the QEEG variables improved outcome prediction, we compared the ROC curves of four models. The first model included the independent predictors of VD, the second included the independent predictors of DTABR, the third included the GCS score, and the fourth included both VD and DTABR. The AUROC for VD and DTABR was 0.896. Comparison of the ROC curves showed that the efficacy of VD and DTABR for the predicting 90-day mortality in patients with PCCI was better than that of the GCS score (AUROC 0.75), VD (AUROC 0.76), or DTABR (AUROC 0.781; all *p* < 0.05). Therefore, the contribution of the final model was significant (Table 3, Figure 4).

**Table 3:** Pairwise comparison of the receiver-operating characteristic curve values.

	**VD**	**DTABR**	**GCS**
Difference between areas	0.135	0.115	0.146
Standard error	0.0651	0.0407	0.0657
95% CI	0.00788–0.263	0.0349–0.194	0.0171–0.275
z statistic	2.081	0.0349–0.194	2.221
Significance level	0.0374	0.0048	0.0263

*P <0.05 for TCD (VD) + QEEG (DTABR) comparison with GCS, VD (independent predictor of TCD), and DTABR (independent predictor of QEEG)*.

*CI, confidence interval; DTABR, (delta + theta)/(alpha + beta) ratio; GCS, Glasgow Coma Scale; QEEG, quantitative electroencephalography; TCD, transcranial Doppler; VD, diastolic flow velocity*.

### Discussion

To the best of our knowledge, this is the first report on the use of TCD-QEEG for examining patients with PCCI. Our study shows that brain function in these patients can be assessed at the bedside with TCD-QEEG. TCD reflects CBF and QEEG reflects neuronal activity; the changes in CBF and neuronal activity are synchronous, and owing to the prospective design, 90-day prognostic information was available. VD in TCD and DTABR in QEEG were the two independent predictors of 90-day mortality. Moreover, after combining the VD and DTABR, the AUROC was 0.896 and superior to that for any single variable. This finding supports the value of TCD-QEEG as a bedside monitoring tool in patients with PCCI.

In previous studies, various parameters have been used to assess the prognosis of patients with PCCI, including age, diabetes mellitus, GCS score, FOUR scores, atrial fibrillation, and ejection fraction (18–20). We found that only the GCS and FOUR scores were associated with mortality. However, they were not independent predictors in the multivariate regression model, possibly because this study included patients who were intubated or had aphasia such that verbal ability could not be assessed in these patients using the GCS. Another possible reason is that some of the patients were awake and the FOUR score is only used for coma patients.

Hypoperfusion determined using large-vessel quantitative magnetic resonance angiography is closely associated with the risk of stroke in patients with symptomatic atherosclerotic vertebral basilar artery occlusive disease (11). The results of TCD in patients with PCCI are a decreasing trend of infratentorial blood flow velocity, indicating that hypoperfusion is an important factor leading to infarction in the posterior circulation (21). TCD is a non-invasive measure of intracranial CBF velocity, which is usually associated with changes in blood flow (22). The spectral waveform derived from TCD is characterized by three components, i.e., VS, VD, and VM, the most clinically relevant of which is VD, especially in intensive care (23). A decrease in cerebral perfusion pressure has an obvious effect on the Doppler waveform, with typical changes that include a decreased diastolic blood flow velocity (16). When cerebral circulation stops, and intracranial pressure starts to increase for whatever reason, there is a decrease in end-diastolic blood flow velocity on TCD (24). A modest increase in VD as opposed to VS was associated with complete recanalization/reperfusion, early neurological improvement, and a favorable functional outcome, suggesting that augmentation of diastolic flow may represent a novel therapeutic reperfusion target (25). We drew a similar conclusion in our TCD study, i.e., that decreases in VS, VD, and VM are significantly correlated with 90-day mortality. The multivariate regression analysis showed that VD was an independent prognostic factor.

When CBF is compromised, changes occur in the metabolic and electrical activities of cortical neurons (26), and QEEG can reflect these changes within seconds. Sheorajpanday et al. found that pdBSI < 0.12 in PCCI was 100% specific for the absence of a recent ischemic lesion, and pdBSI > 0.24 was 100% sensitive for the presence of a recent ischemic lesion, indicating that the pdBSI is an independent predictor of definite stroke in patients presenting with PCCI (15). We found no significant difference in the BSI between our study groups. The reason for this finding may be that BSI is an indicator of the symmetry of bilateral hemisphere damage, and most of the patients with PCCI in our study had double vertebral and/or basilar artery occlusion and bilateral infarcts. However, although there are no reports on QEEG changes after PCCI, many QEEG studies of ischemic stroke in the anterior circulation have confirmed that QEEG correlates well with CBF and brain metabolism. After reviewing the recent studies on the prognosis of patients with cerebral infarction, we found that an increase in relative delta power, DTABR, DAR, and BSI indicated a poor or worsening prognosis (27–31). Good correlations of hemispheric relative delta percentage, spectral edge frequencies, and overall mean frequency with CBF have also been reported (32). In a study that included 13 patients with ischemic cerebral infarction, Finnigan et al. found a statistically significant relationship between the DAR and relative alpha ratio and the 30-day NIH Stroke Scale score (33). Other researchers found that DAR, DTABR, and relative delta could discriminate between patients with acute ischemic stroke and controls (34). In another study, DTABR was the most accurate neurophysiological indicator, with lower relative alpha power and higher DTABR predicting a poor functional outcome and alpha activity showing a negative correlation with the prognosis of stroke (35). Our study showed that the slower frequency band delta power increased, and the more rapid frequency band alpha power decreased after PCCI. Alpha variability, relative delta power, relative alpha power, delta ratio, DAR, spectral entropy, DTABR, median frequency, and peak frequency were all significantly correlated with 90-day mortality. The most significant variables were DTABR and median frequency, and multivariate regression analysis confirmed that DTABR was an independent prognostic factor.

Research on neurovascular coupling dates back hundreds of years. Neurovascular coupling is important for the health of the normal brain (36), and impairment of neurovascular coupling may disrupt regional CBF and metabolic regulation (7). In clinical practice, several methods can be used to assess neurovascular coupling, including a combination of functional MRI or functional near-infrared spectroscopy with EEG (8). TCD combined with QEEG can reflect the relationship between the general metabolism of the brain and CBF. Both modalities are safe, relatively cost-effective, and easy to use. With further advances in science and technology and refinement of equipment, a machine that integrates TCD and QEEG could be developed to allow synchronous monitoring. TCD-QEEG is a very promising tool for monitoring brain function in real-time in the NICU. CT cannot detect PCCI in the first 24 h, and many patients in the NICU are in critical condition with breathing difficulties and are unable to cooperate to the level needed for MRI. Unlike CT and MRI, TCD-QEEG is portable, can show the temporal pattern of neurovascular coupling, and allows a longer monitoring period. TCD-QEEG is a novel neurovascular coupling technique that is non-invasive, can be implemented at the bedside, and can shed light on the synergy between the metabolic and vascular systems. It is likely that TCD-QEEG will soon be available as a synchronous evaluation method.

This study has some limitations. First, it was performed at a single center with a small sample size. Second, we only monitored patients in the acute phase and did not perform dynamic monitoring. In the future, our conclusions need to be verified in a large sample study, and dynamic monitoring is needed to understand the changes in disease progression. Finally, TCD is an operator-dependent technique that requires considerable experience and understanding of the intracranial arterial anatomy. However, the study was performed by an associate professor and an attending physician, which may have contributed to the observed diagnostic accuracy.

In conclusion, our present findings indicate that VD and DTABR are independent prognostic factors for patients with PCCI. TCD combined with QEEG can evaluate the synergy between the metabolic and vascular systems. TCD-QEEG can assess brain function accurately in patients with PCCI and predict the functional prognosis and risk of mortality. This multimodal monitoring technique will provide a better understanding of the coupling mechanisms in the brain affected by PCCI and may lead to improved management of patients with PCCI in intensive care units.

## Data Availability Statement

The original contributions presented in the study are included in the article/supplementary material, further inquiries can be directed to the corresponding author/s.

## Ethics Statement

The studies involving human participants were reviewed and approved by the Ethics Committee of the First Hospital of Jilin University, China (2018-405). The patients/participants provided their written informed consent to participate in this study.

## Author Contributions

YCa contributed to the study conception and design, data collection, analysis and interpretation, and drafting of the manuscript. YCh and YX contributed to the study conception and design, analysis and interpretation of the data, and revision of the final manuscript. XS contributed to the study conception and design, analysis and interpretation of the data, and revision of the manuscript. LW and YQ contributed to the data collection and revision of the manuscript. All authors gave final approval of the version to be published.

## Conflict of Interest

The authors declare that the research was conducted in the absence of any commercial or financial relationships that could be construed as a potential conflict of interest.

## Abbreviations

AUROC: area under the receiver-operating characteristic curve
BSI: brain symmetry index
CBF: cerebral blood flow
DAR: delta/alpha ratio
DTABR: (delta + theta)/(alpha + beta) ratio
EEG: electroencephalography
GCS: Glasgow Coma Scale
NICU: Neurological Intensive Care Unit
PCCI: posterior circulation cerebral infarction
PI: pulsatility index
QEEG: quantitative electroencephalography
ROC: receiver-operating characteristic
TCD: transcranial Doppler
VD: diastolic flow velocity
VM: mean velocity
VS: systolic flow velocity.

## References

[1] Zhou M Wang H Zeng X Yin P Zhu J Chen W. Mortality, morbidity, and risk factors in China and its provinces, 1990-2017: a systematic analysis for the Global Burden of Disease Study 2017. Lancet. (2000) 394:1145–58. 10.1016/S0140-6736(19)30427-1 31248666 PMC6891889

[2] Benjamin EJ Muntner P Alonso A Bittencourt MS Callaway CW Carson AP. Heart disease and stroke statistics-2019 update: a report from the American Heart Association. Circulation. (2019) 139:e56–528. 10.1161/CIR.0000000000000659 30700139

[3] De Marchis GM Kohler A Renz N Arnold M Mono ML Jung S. Posterior versus anterior circulation strokes: comparison of clinical, radiological and outcome characteristics. J Neurol Neurosurg Psychiatry. (2011) 82:33–7. 10.1136/jnnp.2010.211151 20802030

[4] Kim JT Park MS Choi KH Kim BJ Han MK Park TH. Clinical outcomes of posterior versus anterior circulation infarction with low National Institutes of Health Stroke Scale Scores. Stroke. (2017) 48:55–62. 10.1161/STROKEAHA.116.013432 27856952

[5] Lin AQ Shou JX Li XY Ma L Zhu XH. Metabolic changes in acute cerebral infarction: findings from proton magnetic resonance spectroscopic imaging. Exp Ther Med. (2014) 7:451–5. 10.3892/etm.2013.1418 24396424 PMC3881070

[6] Qian HZ Zhang H Yin LL Zhang JJ. Postischemic housing environment on cerebral metabolism and neuron apoptosis after focal cerebral ischemia in rats. Curr Med Sci. (2018) 38:656–65. 10.1007/s11596-018-1927-9 30128875

[7] Guhathakurta D Dutta A. Computational pipeline for NIRS-EEG joint imaging of tDCS-evoked cerebral responses-an application in ischemic stroke. Front Neurosci. (2016) 10:261. 10.3389/fnins.2016.00261 27378836 PMC4913108

[8] Hendrikx D Smits A Lavanga M De Wel O Thewissen L Jansen K. Measurement of neurovascular coupling in neonates. Front Physiol. (2019) 10:65. 10.3389/fphys.2019.00065 30833901 PMC6387909

[9] Abecasis F Oliveira V Robba C Czosnyka M. Transcranial Doppler in pediatric emergency and intensive care unit: a case series and literature review. Childs Nerv Syst. (2018) 34:1465–70. 10.1007/s00381-018-3877-8 29955941

[10] Blanco P Abdo-Cuza A. Transcranial Doppler ultrasound in neurocritical care. J Ultrasound. (2018) 21:1–16. 10.1007/s40477-018-0282-9 29429015 PMC5845939

[11] Amin-Hanjani S Pandey DK Rose-Finnell L Du X Richardson D Thulborn KR. Effect of hemodynamics on stroke risk in symptomatic atherosclerotic vertebrobasilar occlusive disease. JAMA Neurol. (2016) 73:178–85. 10.1001/jamaneurol.2015.3772 26720181 PMC5274634

[12] Astrup J Siesjo BK Symon L. Thresholds in cerebral ischemia-the ischemic penumbra. Stroke. (1981) 12:723–5. 10.1161/01.STR.12.6.723 6272455

[13] O'Gorman RL Poil SS Brandeis D Bollmann S Ghisleni C Lüchinger R. Coupling between resting cerebral perfusion and EEG. Brain Topogr. 26:442–57. 10.1007/s10548-012-0265-7 23160910

[14] Foreman B Claassen J. Quantitative EEG for the detection of brain ischemia. Crit Care. (2012) 16:216. 10.1186/cc11230 22429809 PMC3681361

[15] Sheorajpanday RV Nagels G Weeren AJ De Deyn PP. Quantitative EEG in ischemic stroke: correlation with infarct volume and functional status in posterior circulation and lacunar syndromes. Clin Neurophysiol. (2011) 122:884–90. 10.1016/j.clinph.2010.08.020 20870455

[16] Chen Y Xu W Wang L Yin X Cao J Deng F. Transcranial Doppler combined with quantitative EEG brain function monitoring and outcome prediction in patients with severe acute intracerebral hemorrhage. Crit Care. (2018) 22:36. 10.1186/s13054-018-1951-y 29463290 PMC5820804

[17] Song TJ Kim J Lee HS Nam CM Nam HS Kim YD. Distribution of cerebral microbleeds determines their association with impaired kidney function. J Clin Neurol. (2014) 10:222–8. 10.3988/jcn.2014.10.3.222 25045375 PMC4101099

[18] Hu Y Wang C Yan X Fu H Wang K. Prediction of conscious awareness recovery after severe acute ischemic stroke. J Neurol Sci. (2017) 383:128–34. 10.1016/j.jns.2017.10.034 29246600

[19] Lin SF Chen CI Hu HH Bai CH. Predicting functional outcomes of posterior circulation acute ischemic stroke in first 36 h of stroke onset. J Neurol. (2018) 265:926–32. 10.1007/s00415-018-8746-6 29455362 PMC5878189

[20] Zurcher E Richoz B Faouzi M Michel P. Differences in ischemic anterior and posterior circulation strokes: a clinico-radiological and outcome analysis. J Stroke Cerebrovasc Dis. (2019) 28:710–8. 10.1016/j.jstrokecerebrovasdis.2018.11.016 30501979

[21] Chi HY Hsu CF Chen AC Su CH Hu HH Fu WM. Extracranial and intracranial ultrasonographic findings in posterior circulation infarction. J Ultrasound Med. (2018) 37:1605–10. 10.1002/jum.14501 29193196

[22] Alkhachroum AM Fernandez-Baca Vaca G Sundararajan S DeGeorgia M. Post-subdural hematoma transient ischemic attacks: hypoperfusion mechanism supported by quantitative electroencephalography and transcranial Doppler sonography. Stroke. (2017) 48:e87–90. 10.1161/STROKEAHA.117.016388 28193836

[23] Robba C Cardim D Sekhon M Budohoski K Czosnyka M. Transcranial Doppler: a stethoscope for the brain-neurocritical care use. J Neurosci Res. (2018) 96:720–30. 10.1002/jnr.24148 28880397

[24] Kasapoglu US Haliloglu M Bilgili B Cinel I. The role of transcranial Doppler ultrasonography in the diagnosis of brain death. Turk J Anaesthesiol Reanim. (2019) 47:367–74. 10.5152/TJAR.2019.82258 31572986 PMC6756304

[25] Alexandrov AV Tsivgoulis G Rubiera M Vadikolias K Stamboulis E Molina CA. End-diastolic velocity increase predicts recanalization and neurological improvement in patients with ischemic stroke with proximal arterial occlusions receiving reperfusion therapies. Stroke. (2010) 41:948–52. 10.1161/STROKEAHA.109.577502 20224054

[26] Hossmann KA. Viability thresholds and the penumbra of focal ischemia. Ann Neurol. (1994) 36:557–65. 10.1002/ana.410360404 7944288

[27] Sainio K Stenberg D Keskimäki I Muuronen A Kaste M. Visual and spectral EEG analysis in the evaluation of the outcome in patients with ischemic brain infarction. Electroencephalogr Clin Neurophysiol. (1983) 56:117–24. 10.1016/0013-4694(83)90066-4 6191943

[28] Cuspineda E Machado C Galán L Aubert E Alvarez MA Llopis F. QEEG prognostic value in acute stroke. Clin EEG Neurosci. (2007) 38:155–60. 10.1177/155005940703800312 17844945

[29] Sheorajpanday RV Nagels G Weeren AJ van Putten MJ De Deyn PP. Quantitative EEG in ischemic stroke: correlation with functional status after 6 months. Clin Neurophysiol. (2011) 122:874–83. 10.1016/j.clinph.2010.07.028 20961806

[30] Sheorajpanday RV Nagels G Weeren AJ De Surgeloose D De Deyn PP. Additional value of quantitative EEG in acute anterior circulation syndrome of presumed ischemic origin. Clin Neurophysiol. (2010) 121:1719–25. 10.1016/j.clinph.2009.10.037 20181521

[31] Finnigan S van Putten MJ. EEG in ischaemic stroke: quantitative EEG can uniquely inform (sub-)acute prognoses and clinical management. Clin Neurophysiol. (2013) 124:10–9. 10.1016/j.clinph.2012.07.003 22858178

[32] Tolonen U Sulg IA. Comparison of quantitative EEG parameters from four different analysis techniques in evaluation of relationships between EEG and CBF in brain infarction. Electroencephalogr Clin Neurophysiol. (1981) 51:177–85. 10.1016/0013-4694(81)90007-9 6161792

[33] Finnigan SP Walsh M Rose SE Chalk JB. Quantitative EEG indices of sub-acute ischaemic stroke correlate with clinical outcomes. Clin Neurophysiol. (2007) 118:2525–32. 10.1016/j.clinph.2007.07.021 17889600

[34] Finnigan S Wong A Read S. Defining abnormal slow EEG activity in acute ischaemic stroke: delta/alpha ratio as an optimal QEEG index. Clin Neurophysiol. (2016) 127:1452–9. 10.1016/j.clinph.2015.07.014 26251106

[35] Bentes C Peralta AR Viana P Martins H Morgado C Casimiro C. Quantitative EEG and functional outcome following acute ischemic stroke. Clin Neurophysiol. (2018) 129:1680–7. 10.1016/j.clinph.2018.05.021 29935475

[36] Girouard H Iadecola C. Neurovascular coupling in the normal brain and in hypertension, stroke, and Alzheimer disease. J Appl Physiol. (2006) 100:328–35. 10.1152/japplphysiol.00966.2005 16357086

[37] Cao YT Song XN Wang LJ Qi YJ Chen Y Xing YQ. Transcranial Doppler Combined with Quantitative Electroencephalography Brain Function Monitoring for Estimating the Prognosis of Patients with Posterior Circulation Cerebral Infarction. (2020). Available online at: https://www.researchsquare.com/article/rs-64085/v1 (accessed August 26, 2020). 10.3389/fneur.2021.600985 PMC8165540 34079507

